# Access to effective but expensive treatments: An analysis of the solidarity argument in discussions on funding of medical treatments

**DOI:** 10.1111/bioe.13108

**Published:** 2022-11-07

**Authors:** Sietske A. L. van Till, Jilles Smids, Eline M. Bunnik

**Affiliations:** ^1^ Department of Medical Ethics, Philosophy and History of Medicine, Erasmus MC University Medical Centre Rotterdam Rotterdam The Netherlands

**Keywords:** equality, health care policy, justice, oncology, personalized medicine, solidarity

## Abstract

The development of new effective but expensive medical treatments leads to discussions about whether and how such treatments should be funded in solidarity‐based healthcare systems. Solidarity is often seen as an elusive concept; it appears to be used to refer to different sets of concerns, and its interrelations with the concept of justice are not well understood. This paper provides a conceptual analysis of the concept of solidarity as it is used in discussions on the allocation of healthcare resources and the funding of expensive treatments. It contributes to the clarification of the concept of solidarity by identifying in the literature and discussing four uses of the concept: (1) assisting patients in need, (2) upholding the solidarity‐based healthcare system, (3) willingness to contribute and (4) promoting equality. It distinguishes normative and descriptive uses of the concept and outlines the overlap and differences between solidarity and justice. Our analysis shows that the various uses of the concept of solidarity point to different, even conflicting, ethical stances on whether and how access to effective, expensive treatments should be provided. We conclude that the concept of solidarity has a role to play in discussions on the accessibility and funding of newly approved medical treatments. It requires, for instance, that healthcare policies promote and maintain both societal willingness to contribute to the care of others and the value of providing care to vulnerable patients through public funding.

## 1 BACKGROUND

One of the challenges of modern times is how welfare states should deal with the rising costs of health care while maintaining a sustainable, high‐quality but also equitable healthcare system. New medical treatments are being developed that are tailored to individual patients’ needs by targeting unique genetic or biological characteristics of their disease. Personalized medicine is an increasingly prominent approach in many disease areas, including oncology and rare (genetic) disorders, but is often associated with high costs. High costs increasingly put pressure on universal healthcare systems and are believed to undermine the solidarity on which these systems are built. How should health authorities deal with effective but expensive medical treatments while maintaining solidarity within societies?

An example of an effective but expensive medical treatment is CAR‐T cell therapy, an oncological approach in which patients’ immune cells are genetically modified so that they recognize and attack cancer cells, which costs up to $500,000 per patient.^1^ The cost‐effectiveness of CAR‐T cell therapy in terms of cost per Quality Adjusted Life‐Years (QALY's) strongly depends on the long‐term effectiveness, which, for most indications, is still unclear. In optimistic scenarios, the estimated Incremental Cost‐Effectiveness Ratio (ICER) is around $129,000 per QALY gained.^2^ Another example is Lumacaftor/ivacaftor for Cystic Fibrosis, for which the ICER is estimated between $3,655,352 per QALY gained (base‐case) and $8,480,265 per QALY gained (worst‐case).^3^ In many countries, these costs exceed the ICER cost‐effectiveness threshold for reimbursement through basic health insurance. For example, in the Netherlands, this is set at €80,000 per QALY for severe diseases.^4^ Over the next few years, many new cell and gene therapies are expected to be approved,^5^ each of which may exceed this threshold and may not (immediately) qualify for reimbursement through the healthcare system.

Although individual patients may benefit from newly approved medical treatments, their rising costs may put pressure on society's willingness and ability to collectively fund these treatments. High costs might overstretch solidarity.^6^ The concept of solidarity is deemed very important in healthcare policy in many countries, even if it can be interpreted differently across countries.^7^ In solidaristic healthcare systems, health care is collectively arranged, and all citizens contribute to the care that they themselves and others receive, for instance through (compulsory) health insurance. To protect and sustain solidarity‐based systems, collective healthcare expenses are carefully regulated. Consequently, when medical treatments have limited cost‐effectiveness, they may (very likely) not be reimbursed. In contrast to countries with two‐tier systems with large private markets offering regular medical care, in our country, the Netherlands, there is no, or very limited, possibility to gain access to health care through out‐of‐pocket payments. Treatments that are not reimbursed through mandatory health insurance are commonly not available to Dutch patients. Internationally, it is a topic of discussion whether, if treatments are considered too expensive to be eligible for reimbursement through public funds, doctors may (or should) try and obtain access to such treatments using other means, or whether patients should be allowed to pay for treatments using private funds or, for instance, crowdfunding.^8^ In these discussions, solidarity is invoked to argue that patients should somehow gain access to health care that may provide benefit, but it is also used to argue that they should not, to protect equal process.^9^

The concept of solidarity thus takes centre stage in these discussions, but its meaning is not clear. In this article, we clarify the role(s) the concept of solidarity plays in discussions about funding expensive, effective treatments. To this end, we identify and discuss various uses of the concept of solidarity and the arguments associated with these uses, as found in discussions in the academic literature and public debate on allocating funds or clinical services in health care in general and on funding expensive medical treatments in particular. First, we offer a brief account of the historical development of the concept of solidarity in the bioethical literature, which serves as an analytical framework for the analysis of the various uses of the concept of solidarity. Finally, we discuss how solidarity as an ethical concept may contribute to discussions about funding expensive treatments.

## 2 CONCEPT OF SOLIDARITY IN BIOETHICAL LITERATURE

For a long time, solidarity was underexposed as a concept in the bioethical literature and considered elusive,^10^ but in the past two decades, it has gained more attention. Prainsack & Buyx have given a significant impulse to this discussion and offered the following working definition of solidarity:

shared practices reflecting a collective commitment to carry ‘costs’ (financial, social, emotional, or otherwise) to assist others.^11^

Building on their work and that of others, we point out five dimensions and distinctions within various conceptions of solidarity (see Table 1).

**Table 1:** Dimensions and distinctions within conceptions of solidarity

Tiers	Tier 1: Interpersonal level Tier 2: Group practices Tier 3: Contractual and legal manifestations
Status	Descriptive Normative ‐Intrinsic/freestanding ‐Instrumental/auxiliary
Motivational basis	Rational (planned and consensual) ‐Common good ‐Interest solidarity Nonrational (social sense of the common) ‐Constitutional solidarity ‐Recognition of sameness/similarity ‐Humanitarian solidarity
Relationship with justice	Corresponding Auxiliary (‘putty’) Independent

First, solidarity must be reflected in actions, and thus, it is necessary nor sufficient for individuals to have an emotion, such as empathy, to be characterized as solidaristic. Actions or practices must ensue.^12^

Second, Prainsack and Buyx stated that solidarity can manifest itself on various levels or tiers: (1) the interpersonal level (‘willingness to carry costs to assist others with whom a person recognises sameness or similarity in at least one relevant respect’), (2) group practices (‘collective commitment to carry costs to assist others (who are all linked by means of a shared situation or cause)’) and (3) contractual and legal manifestations (‘contractual or other legal norms’).^13^ Solidarity‐based healthcare systems involve legal obligations for citizens to contribute financially, for instance, which are a tier 3 manifestation of solidarity. Tier 3 solidarity may involve ‘income solidarity’ and ‘risk solidarity’, meaning that more affluent citizens contribute to the health care of less affluent citizens, and healthier citizens contribute to the health care of the ill, often by making (mandatory) financial contributions. The higher tiers of solidarity are often supported by the lower tiers; healthcare systems (tier 3) can be supported by willingness among individual citizens to contribute (tier 1) and by group norms that propagate mutual support (tier 2). However, lower tiers do not necessarily develop into higher tiers.

Third, there has been an ongoing discussion in the ethical literature about the moral relevance of solidarity. The concept can be used both normatively and descriptively.^14^ When used descriptively, solidarity may refer to the factual strength of relationships and mutual involvement within a community, which can be examined empirically. Used normatively, solidarity can be seen either as an intrinsic or freestanding value, which for example can be defined as ‘the unselfish dedication to a fellow human being who is in need’,^15^ but also as an auxiliary or instrumental value that relies, for its normative force, on underlying principles of justice or beneficence.^16^

Fourth, different accounts are given of the motivational forces underlying solidaristic actions. Roughly, rational accounts of solidarity can be distinguished from nonrational accounts. Rational solidarity is explained as built either on self‐interest or on a joint commitment to achieve a common goal (e.g., population health).^17^ Willingness to contribute to the healthcare system as motivated by self‐interest is known as interest solidarity.^18^ But even if the motivation lies in the common good, solidarity may be considered rational when, for instance, it is focused on the maximization of population health outcomes or limited by the requirement of cost‐effectiveness. There are several accounts of nonrational solidarity that partly overlap. ‘Constitutional’ accounts of solidarity focus on the fostering of communitarian bonds in groups or communities, whose identities are formed by shared values, projects, aims and understandings.^19^ Some accounts are built on the recognition of similarity or sameness.^20^ Normatively, they imply ‘standing up for each other because one recognises one's own fate in the fate of the other’.^21^ They emphasize people's responsibilities for members of the same group (e.g., residents of a country or community) and the importance of mutual support in opposing collective threats.^22^ Humanitarian solidarity builds on the recognition of the vulnerability of others and requires a ‘willingness to protect those human persons whose existence is threatened by circumstances beyond their own control, particularly natural fate or unfair social structures’.^23^

Fifth and finally, solidarity seems to have an important relationship with the concept of justice, which is not always explicated, and the concepts are sometimes used interchangeably, taken to be identical, or at least seen as corresponding.^24^ However, solidarity and justice are distinct concepts, and it is important to be clear about their distinct roles in normative theorizing about health care.^25^ Whereas solidarity can best be seen as belonging to the axiological realm, justice is clearly a deontic concept.^26^ The willingness to carry cost at an interpersonal level, which is fundamental to solidarity, qualifies as morally valuable or as a moral virtue. Justice, on the contrary, is a duty‐ and rights‐based concept, focusing on the moral obligations that individuals have, regardless of whether they are in fact motivated to act on such obligations. This distinction is crucial for grounding the moral obligations of citizens to uphold their healthcare system. Bayertz observes that it is inadequate to conceptualize the legally enforced financial redistribution that is part of the welfare state, as an expression of interpersonal solidarity.^27^ The anonymous and distant relations in which moral obligations arise between citizens to maintain public systems are very different from the direct communal relations in families and local neighbourhoods that naturally give rise to solidaristic moral motivations. Accordingly, Bayertz argues, Rawls’ justification for his well‐known principles of justice^28^ does not presuppose a community, but a group of rationally self‐interested, free and equal individuals, who are contracting behind a veil of ignorance. Most theories of justice similarly hold that the duty of citizens to pay taxes or insurance premiums to ensure decent healthcare for all is, ultimately, grounded in citizens’ equal moral worth.^29^ Solidarity often has a supererogatory character. Another difference between solidarity and justice is that solidaristic appeals for aid can be endless, whereas justice‐based claims can often be limited clearly, using criteria such as cost‐effectiveness and disease severity. Consequentialist considerations of cost‐effectiveness allow health agencies to develop clear procedures for priority setting and limiting healthcare expenses, to maximize health gains given fixed budgets. This focus on the maximization of health gains is mitigated by societies’ higher willingness to pay for those who are more severely ill, which generally is seen as having a justice‐based rationale.^30^

Given these conceptual distinctions and associated moral obligations, most authors argue that justice takes normative precedence over solidarity when it comes to justifying public healthcare decision‐making. Solidarity, however, still has a clear auxiliary role to play in helping to realize the more fundamental requirements of justice:^31^ when individuals recognize similarities with other citizens and shared interest in decent health care, they may be more willing to contribute to a just (universal) care system.

The different conceptions of solidarity just described complicating a good understanding and fruitful application of this concept to specific cases. Yet, our analysis of the literature has uncovered several distinctions and considerations (see Table 1), which will be used in the next section to analyse the various uses of the concept of solidarity in practice.

## 3 FOUR USES OF SOLIDARITY IN THE CONTEXT OF FUNDING OF EXPENSIVE TREATMENTS

We identified four different uses of the concept of solidarity in the discussions on allocating funds and resources generally in health care and on funding expensive treatments in particular: (1) assisting patients in need, (2) upholding the solidarity‐based healthcare system, (3) willingness to contribute and (4) promoting equality.

### 3.1 Assisting patients in need

First, we note that solidarity is used in discussions to refer to a moral obligation to offer *assistance to people who are in need*.^32^ This is perhaps most in line with the meaning of solidarity in everyday discourse. Expensive treatments, such as CAR‐T cell therapy, are often intended for patients with serious or life‐threatening illnesses, who are in acute need and sometimes have no other options for treatment. These patients can gain access to potentially life‐saving treatments through the collective commitment of fellow citizens to contribute financially to their costs. Many citizens, when confronted with seriously ill patients, will feel a sense of sympathy and an urge to help them. In this use of solidarity, both descriptive and normative elements are at play. First, solidarity can be used to describe an individual's factual, emotional response, or a human predisposition to help patients who are in acute need.^33^ This psychological response is referred to by Jonsen as the ‘rule of rescue’ and is especially strong when ‘identified’ individuals are in life‐threatening situations and when there are lifesaving (treatment) options available.^34^ It is characterized by disregarding for the costs involved. Solidarity can also be used more normatively, providing an argument for collective reimbursement of medical care, even if it is (very) expensive. Humanitarian solidarity, for instance, focuses on the protection of vulnerable others and includes a commitment to assisting those in need.^35^ It puts forward a moral *obligation* to help patients in need of medical treatment. As patients requiring expensive treatments are often severely ill and thus most in need, they should be made available, regardless of the cost. An example of this first use of solidarity is the following:

Even when the decision process is democratic and transparent, however, each time a drug is excluded from the publicly funded package, there is a reduction in solidarity with those patients who would have benefited from its inclusion.^36^

The urge to help vulnerable patients (whether emotionally or normatively driven) at the individual (tier 1) and group (tier 2) levels serves as an important foundation for institutionalized manifestations of solidarity in collectively funded healthcare systems (tier 3). At first sight, this use of solidarity provides an argument for collective funding of medical treatments, even if they are expensive.

### 3.2 Upholding the solidarity‐based healthcare system

The concept of solidarity is often used to express the concern that the sustainability of universal healthcare systems is being threatened by the rising prices of medical treatments.^37^ Many resource‐rich countries have universal, solidarity‐based healthcare systems in place that rely on income solidarity and risk solidarity to ensure that everybody has equal access to a decent level of health care.^38^ Solidarity‐based healthcare systems are contractual and legal manifestations of solidarity (tier 3) in which all citizens (must) actively contribute to the care of others and themselves,^39^ demonstrating both shared (rational) interest and (constitutional) commitment to shared values, such as equality.^40^ Given that resources are inevitably finite and that it is impossible to collectively fund all the care that is desired, healthcare costs must somehow be contained. Therefore, medical treatments that are not cost‐effective or simply too expensive might not be eligible for reimbursement in a solidarity‐based healthcare system.^41^ First of all, the provision of less cost‐effective care brings the risk of displacement of other, more cost‐effective care, leading to worse health outcomes for the group, which may run counter to the aims of risk solidarity. After all, displacement may occur directly, for example, through lack of staff and bed capacity in hospitals, but also indirectly, for example, through reduced funding for other healthcare activities, including preventive care.^42^ As a result of displacement, fewer health benefits would be achieved across the population. Suboptimal use of healthcare resources is a problem when rational solidarity is assumed: when citizens contribute financially to a healthcare system because they expect health benefits in return when they themselves are in need. It is also a problem when, for instance, constitutional solidarity is assumed, as higher premiums put a strain on citizens’ ability and willingness to continue to contribute financially to the healthcare system for the benefit of others. We will discuss below (use 3) how, when the solidaristic healthcare system becomes too demanding, a sense of sameness may dissolve and ultimately, constitutional solidarity may collapse.

This second use of solidarity in discussions on the funding of expensive treatments thus refers to the importance of careful consideration of the cost‐effectiveness of medical treatments to maintain healthcare systems in which all citizens have equal access to health care. With cost containment as its central consideration, this second use differs from the first, assisting patients in need, regardless of the cost. In fact, it may even be diametrically opposed to it. Arguably, upholding a solidarity‐based system is a matter of distributive justice, which, as said, is considered a stronger ethical requirement than solidarity (use 1), even if the two are seen as independent moral values. To ensure that access to health care is—and continues to be—fair and just, collective funding of medical treatments that are effective but considered too expensive, may not be acceptable. Thus, in a solidarity‐based system, a ‘solidarity‐inspired rule of rescue does not have a place’.^43^

It follows that to safeguard the second use of solidarity, collective funds should not be used to offer medical treatments that are not or are insufficiently cost‐effective. Although many may wish to fund expensive treatments for individual patients based on an imperative to assist patients in need, this second use of solidarity offers an argument against doing so.

### 3.3 Willingness to contribute

Some uses of solidarity are less normative and provide less of an answer to the question of whether expensive treatment should be publicly funded. Solidarity can be used descriptively, describing the actual willingness within societies to contribute to the care of others.^44^ This willingness can be examined empirically and forms an indication of the strength of social ties in societies. Monitoring of (descriptive) solidarity is important especially in liberal democracies with mandatory health insurance, to ensure continued public support and help sustain the system (solidarity at tier 3). As Prainsack and Buyx point out, reimbursement policies must often be supported by the lower tiers of solidarity (tier 1 and 2) to be sustainable.^45^ The use of solidarity as the level of willingness within society to contribute to a solidarity‐based healthcare system is perhaps less often put forward in academic literature, but it is especially prominent in the public debate in our country, the Netherlands, and has a prominent role in reimbursement decision‐making.^46^ Do people support reimbursement of very expensive medical treatments? Ter Meulen warns against a current weakening of social ties, which may result in decreasing willingness to contribute.^47^ When people are asked, or rather obliged, to contribute more and more of their income to the care of others, their willingness to contribute to the healthcare system might be overstretched. Kolers writes: ‘If solidarity is the putty of justice, then, it has the following feature […]: it has an upper limit in terms of how much strain it can bear’.^48^ States cannot make overly demanding solidarity appeals to individuals, as this will put too much pressure on this reciprocal bond between citizens. It might result in what is referred to in the literature as ‘scrutiny’,^49^ a social dynamic in which citizens no longer have an unconditional willingness to contribute to the care of others. Instead, health care is allocated only after an (invasive) checking and assessing whether individuals meet the eligibility criteria for the health care they request and whether these individuals are ‘solidarity‐worthy’. As a result, patients are criticized or held accountable for their healthcare needs and expenditures. In a society governed by scrutiny, a sense of sameness is lost, which undermines constitutional forms of solidarity that are characterized by an unconditional (or less conditional) willingness to assist others. Instead, it is felt that individuals who make claims to collectively funded healthcare resources should reciprocate by taking responsibility for limiting their demands for health care and helping relieve the pressure they put on the community. For instance, in public discussions in the Netherlands, calls for limits to solidarity are sometimes linked to the (minority) view that people with lifestyle related diseases should bear responsibility for their healthcare demands.^50^

The same phenomenon can be observed in public discussions on the funding of medical treatments for patients with rare (genetic) diseases, which are often costly.^51^ Rational conceptions of solidarity may likewise lead to scrutiny:^52^ citizens may become less solidaristic with patients who need care that they will probably not claim themselves. People may feel that they have no interest in contributing to care for patients with rare diseases, as rare diseases affect a relatively small population and the chances that people themselves will develop these diseases are negligible or nonexistent. Collective funding of very expensive treatments, such as CAR‐T cell therapy, might potentially undermine citizens’ willingness to make financial contributions to the (very expensive) care of (very few) others. In this descriptive conception of solidarity, in sum, collective funding of medical treatments that are considered too expensive is undesirable as it may undermine society's continued willingness to contribute to a solidary‐based system.

### 3.4 Promoting equality

While the first three uses refer mainly to the allocation of public funds, the fourth concerns equality of access to medical treatments for individual patients when collective funding is *not* available. This use of solidarity holds that patients should not have access to medical treatments that are too expensive or not cost‐effective, even if they paid for themselves. As health is of special importance to everyone, everyone should have access to necessary health care of the same, decent, quality.^53^ Focus group studies have shown, for example, that Dutch citizens do not consider it solidaristic if only the more affluent can gain access to new technologies while the less affluent cannot: especially access to potentially life‐saving treatments should not depend on the ability to pay.^54^ In a solidarity‐based healthcare system, no exceptions are to be made for patients with higher incomes. This use of solidarity can have implications for both higher and lower tiers. First, on the level of the healthcare system (tier 3), it refers to equal treatment and access, even if this does not lead to optimal health outcomes for individual patients. Offering medical care through the private sector and allowing patients to pay out of pocket are thus not in line with this use of solidarity. This is especially so if the public healthcare system does not provide a decent level of care and the less affluent has no access to the medical care they need.^55^ If expensive treatments such as CAR‐T cell therapy are not reimbursed using collective funds, few people will be able to pay for them out of pocket. Only the (very) rich, and perhaps those who have the capacity to organize successful fundraising campaigns, will be able to access these treatments. This introduces divisions between individual patients and groups of patients along socio‐economic lines, associated with diverging opportunities to benefit from these treatments. Failure to ensure equal access to health care is seen as a violation of solidarity.

Second, solidarity on the group level (tier 2) manifests as a feeling of ‘being in the same boat’ and a moral commitment to stand beside one another.^56^ Individual patients with the financial means to purchase medical treatments that are not reimbursed through the healthcare system, it suggests, should forego treatment based on solidarity with other patients with the same disease, who do not have such financial means, a phenomenon referred to as ‘self‐rationing’.^57^ Here, solidarity occurs on the level of decisions made by individual patients, who are themselves in need of medical treatment, for the good of the patient group (that is, to safeguard equal access). Patients recognize sameness in one another and share experiences of illness and a common need for a nonreimbursed treatment. In patient associations, they gather and draw hope and support from each other.^58^ Gould refers to this use as ‘network solidarity’, in which individuals support each other and are stronger when facing suffering together.^59^ As solidarity should not be limited to feelings of mutual empathy, but must also be reflected in actions, patients should be consistent and keep standing together for equal access to potentially beneficial treatments. Buying one's own medical treatment, while knowing that one's fellow patients do not have access to the same treatment, based on their inability to pay, would contradict this use of solidarity.

Solidarity as promoting equality implies a form of ‘levelling down’: if not everyone can have access to these treatments, no one should have access. According to the first use of solidarity we discussed, society should do as much as possible to provide care to patients in need (including, possibly, allowing patients to use private funds). By contrast, this fourth use implies that inequality should not occur and that access to important resources should not be made dependent on income. From this use of solidarity, it follows that inequalities in access to medical treatments should not be allowed and that when treatments are not reimbursed through the public healthcare system, patients should not (be able to) pay for treatments using private funds.

## 4 DISCUSSION

While solidarity is considered ‘a fundamental social value that ought to be nourished and cultivated’, it is also seen as ‘too abstract a notion’ to be used to articulate whether or not patients should have access to effective but very expensive treatments.^60^ To help further this discussion, we have distinguished four uses of the concept of solidarity in discussions about the allocation of healthcare resources and the funding of effective but expensive medical treatments: (1) assisting patients in need, (2) upholding the solidarity‐based healthcare system, (3) willingness to contribute and (4) promoting equality. These four uses involve substantially different interpretations and applications of ‘solidarity’. We will now discuss tensions and relations of mutual support between the four uses of ‘solidarity’, put forward the potential conflict between the concepts of justice and solidarity in discussions on public funding of medical treatments and carve out a role for solidarity in discussions on the funding of expensive medical treatments.

An important finding of our investigation is that there are both relations of mutual support between the four uses of solidarity and tensions between them. Notably, unlimited solidarity as *assisting patients in need* (use 1) leads to ever‐increasing healthcare costs that endanger upholding solidarity‐based healthcare systems (use 2) and could undermine citizens’ willingness to contribute to the care of others (use 3). The urge to assist our fellow citizens in need is especially felt when expensive but effective treatments are available to socially visible groups of patients with severe diseases. In such instances, the rule of rescue comes into play.^61^ For example, in the Netherlands in 2017, health authorities gave in to societal pressure, in response to reports of patients’ plight in the media, to fund the very expensive treatment Lumacaftor/ivacaftor, which was originally deemed insufficiently cost‐effective to be eligible for reimbursement through health insurance.^62^ The impulse is understandable; it is difficult to stand idly by when fellow citizens’ lives are visibly threatened and potentially effective treatments are available.^63^ This may be especially difficult for patients’ families, who are emotionally involved, and healthcare professionals, who are responsible for acting in the best interest of patients. However, it is unclear to what extent the rule of rescue is morally defensible.^64^ If health authorities act upon the solidaristic (tier 1) impulse, they would contradict and undermine their own methods and processes for decision‐making that were developed to uphold an efficient and cost‐effective healthcare system (tier 3). Prioritizing solidarity (use 1) could threaten the sustainability of the healthcare system (use 2) and the fair distribution of healthcare resources (e.g., through displacement of other medically necessary care). The concept of justice provides clear guidance in reimbursement decision‐making by fairly weighing the needs of individual patients against the necessity of guaranteeing decent health care for the entire population. The costs of Lumacaftor/ivacaftor and CAR‐T cell therapy, for instance, are extremely high, while the (long‐term) health benefits are uncertain. Justice and solidarity (as in upholding a just healthcare system (use 2)) may thus require that sometimes (too) expensive or insufficiently cost‐effective medical treatments are not always reimbursed to patients in need.

Solidarity is also employed in lines of argumentation regarding private funding of expensive treatments, pointing in different directions. For example, in the Netherlands, not all CAR‐T cell therapies are publicly funded (yet), which leads to concerns about their accessibility for patients with variable financial resources. Crowdfunding campaigns, in which individuals contribute informally to financing of the care of other citizens, can be seen as exemplary acts of solidarity, in which fellow citizens in need are helped by the community (use 1). However, such acts can also have negative consequences. Allowing patients to pay for medical treatments using private funds introduces inequality and thus runs counter to solidarity as equality between patients (use 4). Out‐of‐pocket payment for health care does not fit within solidarity‐based healthcare systems, in which every citizen should have equal access to healthcare. Moreover, it may degrade solidarity among patients: more affluent patients must not leave their less affluent fellow patients behind. In sum, also in response to the question whether private funding of expensive treatments should be allowed, the concept of solidarity can be used to provide different, opposing answers. Here, however, it is not clear whether these tensions can be resolved with the help of theories of justice. Prohibition of out‐of‐pocket payment would amount to levelling down,^65^ which is difficult to justify. Even most egalitarians are pluralists who agree that equality may be trumped by other values, including health and wellbeing of patients. Further work should thus focus on the analysis of the dilemma of out‐of‐pocket payment from the perspective of justice and other ethical concepts, including beneficence and liberty.

Solidarity does have an important role to play in decision‐making about public funding of expensive treatments in universal healthcare systems. We envision the following role for the concept of solidarity. In its descriptive sense, seen as the strength of the factual social cohesion within society (use 3), solidarity is necessary to establish broad support for public healthcare systems. Often, self‐interest is insufficient to motivate citizens to support a public healthcare system because they frequently have to contribute to care they (probably) will not need themselves. Rather, the motivational basis to contribute to the care of patients (use 3) and to support collective funding of health care in general (use 2) lies in strong bonds of constitutional and humanitarian solidarity among citizens, in which others are recognized as equals. The auxiliary role of solidarity is therefore indispensable: a just healthcare system *needs* solidarity to realize its goals. Willingness to perform solidaristic acts is therefore not only of a noncommittal and supererogatory significance, but may be seen as morally required to support a just healthcare system. Thus, in our view, solidarity may complement and support the concept of justice.

Finally, it will be clear from our investigation and discussion that a fruitful employment of the concept of solidarity in bioethical discussions requires being explicit about the sense in which it is used.

## 5 CONCLUSION

We have discussed four uses of solidarity in discussions about the allocation of healthcare resources and the public funding of expensive treatments, ranging from the commitment to help patients in need to the requirement to maintain a just healthcare system and to ensure equal access to basic health care. Different uses of solidarity may provide conflicting answers to the question whether or not effective but expensive medical treatments should be covered using public funds. Although solidarity (assisting patients in need) can provide normative support for public funding of expensive treatments, it does not necessarily require such funding when it conflicts with requirements of justice or when it may undermine a public healthcare system. And while allowing individual patients to pay for treatments that are not (yet) reimbursed may help alleviate their needs, it introduces inequality and may deteriorate solidarity between patients. The concept of solidarity is prominent in discussions on the funding of expensive medical treatments and rightly so. Most importantly, it refers to the importance of fostering willingness among fellow citizens to contribute to the care of others and to support a just healthcare system. The rising costs of medical treatments may jeopardize this willingness and thereby the sustainability of public healthcare systems. In decision‐making about public funding of medical treatments, the concept of justice takes priority and may support decisions against public funding of medical treatments that are insufficiently cost‐effective. It is not clear, however, whether, in public healthcare systems, allowing patients to pay for treatments using private funds conflicts with justice requirements. Further study should elucidate this.

## CONFLICT OF INTEREST

The authors declare no conflict of interest.

## Footnotes

1 American Cancer Society. (2021). *CAR T‐cell therapy and its side effects*. https://www.cancer.org/treatment/treatments-and-side-effects/treatment-types/immunotherapy/car-t-cell1.html; Hernandez, I., Prasad, V., & Gellad, W. F. (2018). Total costs of chimeric antigen receptor T‐cell immunotherapy. *JAMA Oncology, 4*(7), 994–996.

2 Lin, J. K., Muffly, L. S., Spinner, M. A., Barnes, J. I., Owens, D. K., & Goldhaber‐Fiebert, J. D. (2019). Cost effectiveness of chimeric antigen receptor T‐cell therapy in multiply relapsed or refractory adult large B‐cell lymphoma. *Journal of Clinical Oncology*, *37*(24), 2105–2119.

3 Sharma, D., Xing, S., Hung, Y. T., Caskey, R. N., Dowell, M. L., & Touchette, D. R. (2018). Cost‐effectiveness analysis of lumacaftor and ivacaftor combination for the treatment of patients with cystic fibrosis in the United States. *Orphanet Journal of Rare Diseases, 13*(1), 172.

4 Council for Public Health and Health Care. (2006). *Advies ‘Zinnige en duurzame zorg’* (pp. 32–35). https://www.raadrvs.nl/documenten/publicaties/2006/06/07/zinnige-en-duurzame-zorg

5 American Cancer Society. (2020). *Precision or personalized medicine*. https://www.cancer.org/treatment/treatments-and-side-effects/treatment-types/precision-medicine.html

6 Adema, W. (2015). *Te dure geneesmiddelen ondermijnen solidariteit*. https://www.skipr.nl/blog/te-dure-geneesmiddelen-ondermijnen-solidariteit/; Mandemaker, A. (2019). *Nieuwe topman CZ: ‘Solidariteit zorgstelsel in gevaar’*. https://www.ed.nl/eindhoven/nieuwe-topman-cz-solidariteit-zorgstelsel-in-gevaar%7Ea4299435/?referrer=https%3A%2F%2Fwww.google.nl%2F

7 Saltman, R. B. (2015). Health sector solidarity: A core European value but with broadly varying content. *Israel Journal of Health Policy Research, 4*, 5.

8 Fenton, E. (2011). Mind the gap: Ethical issues of private treatment in the public health system. *New Zealand Medical Journal, 124*(1334), 89–96; Jackson, E. (2010). Top‐up payments for expensive cancer drugs: Rationing, fairness and the NHS. *Modern Law Review, 73*(3), 399–427.

9 Kerr, A., Chekar, C. K., Swallow, J., Ross, E., & Cunningham‐Burley, S. (2021). Accessing targeted therapies for cancer: self and collective advocacy alongside and beyond mainstream cancer charities. *New Genetics and Society, 40*(1), 112–131; Saltman, op. cit. note 7, p. 5.

10 Prainsack, B., & Buyx, A. (2012). Solidarity in contemporary bioethics—Towards a new approach. *Bioethics, 26*(7), 343–350.

11 Prainsack, B., & Buyx, A. (2011). *Solidarity: Reflections on an emerging concept in bioethics* (pp. 46–50). Nuffield Council on Bioethics. https://www.nuffieldbioethics.org/assets/pdfs/Solidarity-report.pdf

12 Ibid.

13 Ibid.

14 Ibid: 36.

15 Ter Meulen, R. (2015). Solidarity and justice in health care. A critical analysis of their relationship. *Diametros, 43*, 1–20.

16 Kolers, A. (2021). What does solidarity do for bioethics? *Journal of Medical Ethics, 47*(2), 122–128; Prainsack, B., & Buyx, A. (2017). *Solidarity in biomedicine and beyond* (pp. 73–80). Cambridge University Press.

17 Dawson, A., & Verweij, M. (2012). Solidarity: A moral concept in need of clarification. *Public Health Ethics, 5*(1), 1–5.

18 Ter Meulen, op. cit. note 15, p. 5.

19 Dawson & Verweij, op. cit. note 17, pp. 1–5.

20 Prainsack & Buyx, op. cit. note 11, pp. 46–50.

21 Jaeggi, R. (2001). Solidarity and indifference. In R. Ter Meulen, W. Arts, & R. Muffels (Eds.), *Solidarity in health and social care in Europe. Philosophy and medicine* (Vol. 69, pp. 287–308). Springer.

22 Gould, C. C. (2018). Solidarity and the problem of structural injustice in healthcare. *Bioethics, 32*(9), 541–552.

23 Ter Meulen, op. cit. note 15, p. 4.

24 Council for Public Health and Health Care. (2007). *Advies ‘Rechtvaardige en duurzame zorg’* (pp. 17–24). Den Haag. https://www.raadrvs.nl/documenten/publicaties/2007/10/17/rechtvaardige-en-duurzame-zorg

25 We thank an anonymous reviewer for urging us to provide more clarity on the distinctions between solidarity and justice.

26 Prainsack & Buyx, op. cit. note 16, p. 77.

27 Bayertz, K. (1999). Four uses of “solidarity”. In K. Bayertz (Ed.), *Solidarity. Philosophical studies in contemporary culture* (pp. 3–28). Springer.

28 Rawls, J. (2005). *A theory of justice*. The Belknap Press of Harvard University Press.

29 Schramme, T. (2018). *Theories of health justice: Just enough health*. Rowman & Littlefield International.

30 Daniels, N. (2001). Justice, health, and healthcare. *American* Journal of *Bioethics*, *1*(2), 2–16.

31 Prainsack & Buyx, op. cit. note 16, p. 79; Houtepen, R., & Ter Meulen, R. (2000). New types of solidarity in the European welfare state. *Health Care Analysis, 8*(4), 329–340; Kolers, op. cit. note 16, pp. 122–128.

32 Condit, D. P. (2016). Catholic social teaching: Precepts for healthcare reform. *Linacre Quarterly, 83*(4), 370–374; Gheaus, A. (2017). Solidarity, justice and unconditional access to healthcare. *Journal of Medical Ethics, 43*(3), 177–181; Ter Meulen, R. (2016). Solidarity, justice, and recognition of the other. *Theoretical Medicine* and *Bioethics, 37*(6), 517–529; Ter Meulen, R., & Maarse, H. (2008). Increasing individual responsibility in Dutch health care: Is solidarity losing ground? *Journal* of *Medicine* and *Philosophy, 33*(3), 262–279.

33 Verweij, M. (2015). How (not) to argue for the rule of rescue claims of individuals versus group solidarity. In G. Cohen, N. Daniels, & N. Eyal (Eds.), *Identified versus statistical victims. An interdisciplinary perspective* (pp. 137–149). Oxford University Press.

34 Jonsen, A. R. (1986). Bentham in a box: Technology assessment and health care allocation. *Law, Medicine, and Health Care, 14*(3–4), 172–174.

35 Ter Meulen, op. cit. note 15, p. 5.

36 Saltman, op. cit. note 7, p. 5.

37 Buyx, A., & Prainsack, B. (2012). Lifestyle‐related diseases and individual responsibility through the prism of solidarity. *Clinical Ethics, 7*(2), 79–85; Ter Meulen, R. H. (1995). Limiting solidarity in the Netherlands: a two‐tier system on the way. *Journal* of *Medicine* and *Philosophy, 20*(6), 607–616; Verweij, op. cit. note 33, pp. 137–149.

38 Busse, R., Blümel, M., Knieps, F., & Bärnighausen, T. (2017). Statutory health insurance in Germany: A health system shaped by 135 years of solidarity, self‐governance, and competition. *Lancet, 390*(10097), 882–897; Ten Have, H. A. M. J., Ter Meulen, R. H. J., & van Leeuwen, E. (2009). *Medische ethiek* (3rd ed., pp. 126–129). Bohn Stafleu van Loghum.

39 Prainsack & Buyx, op. cit. note 10, pp. 343–350.

40 Dawson & Verweij, op. cit. note 17, pp. 1–5.

41 Council for Public Health and Health Care, op. cit. note 4, pp. 32–35.

42 Verweij, op. cit. note 33, pp. 137–149.

43 Verweij, op. cit. note 33, pp. 137–149.

44 Prainsack & Buyx, op. cit. note 11, p. 36.

45 Prainsack & Buyx, op. cit. note 11, pp. 46–50.

46 Council for Public Health and Health Care, op. cit. note 24, p. 94; Wijma, S. (2018). *Solidariteit*. Dutch National Health Care Institute. https://www.zorginstituutnederland.nl/actueel/weblog/weblogberichten/2018/solidariteit

47 Ter Meulen, R., & van der Made, J. (2000). The extent and limits of solidarity in Dutch health care. *International Journal of Social Welfare, 9*, 251.

48 Kolers, op. cit. note 16, pp. 125–126.

49 Houtepen & Ter Meulen, op. cit. note 31, pp. 330–331.

50 Kooiker, S., de Klerk, M., Ter Berg, J., & Schothorst, Y. (2012). *Meebetalen aan de zorg* (pp. 36–37). The Netherlands Institute for Social Research (SCP); The Netherlands Institute for Social Research (SCP). (2019). *Burgerperspectieven 2019*|*2* (pp. 45–47). https://www.scp.nl/actueel/nieuws/2019/06/28/de-zorg-een-grote-maatschappelijke-kwestie-voor-velen-een-persoonlijke-zorg-vooral-voor-wie-het-financieel-lastig-heeft

51 Ten Have, H. A. M. J., et al., op. cit. note 38, pp. 126–129.

52 Ter Meulen, op. cit. note 15, pp. 1–20.

53 Feiring, E., & Wang, H. (2018). Rationing cancer treatment: a qualitative study of perceptions of legitimate limit‐setting. *BMC Health Services Research, 18*(1), 342; Gould, op. cit. note 22, pp. 541–552; Kooiker, S., et al., op. cit. note 50, pp. 40–42; Verweij, op. cit. note 33, pp. 137–149.

54 Kooiker, S., et al., op. cit. note 50, pp. 40–42.

55 Gould, op. cit. note 22, pp. 541–552.

56 Dawson, A., & Jennings, B. (2012). The place of solidarity in public health ethics. *Public Health Review, 34*(1), 65–79; Prainsack & Buyx, op. cit. note 10, p. 348.

57 Fleck, L. M. (2022). Precision medicine and the fragmentation of solidarity (and justice). *Medicine, Health Care and Philosophy, 25*(2), 191–206.

58 Mascalzoni, D., Petrini, C., Taruscio, D., & Gainotti, S. (2017). The role of solidarity(‐ies) in rare diseases research. *Advances in Experimental Medicine and Biology, 1031*, 589–604.

59 Gould, op. cit. note 22, pp. 541–552.

60 Fleck, op. cit. note 57, pp. 191–206.

61 Jonsen, op. cit. note 34, pp. 172–174.

62 Dutch National Health Care Institute. (2017). *Orkambi®: van beoordeling tot vergoeding*. https://www.zorginstituutnederland.nl/publicaties/publicatie/2017/10/26/orkambi-van-beoordeling-tot-vergoeding

63 Hadorn, D. C. (1991). Setting health care priorities in Oregon. Cost‐effectiveness meets the rule of rescue. *JAMA, 265*(17), 2218–2225.

64 McKie, J., & Richardson, J. (2003) The rule of rescue. *Social Science & Medicine*, *56*(12), 2407–2419.

65 Eyal, N. (2013). Leveling down health. In N. Eyal, S. A. Hurst, O. F. Norheim, & D. Wikler (Eds.), *Inequalities in health: Concepts, measures, and ethics* (pp. 200–213). Oxford University Press Inc.

